# 贝林妥欧单抗治疗成人急性B淋巴细胞白血病16例临床分析

**DOI:** 10.3760/cma.j.cn121090-20240611-00219

**Published:** 2025-03

**Authors:** 之茵 刘, 苏江 张, 泽莹 严, 海敏 孙, 玉宝 陈

**Affiliations:** 上海交通大学医学院附属瑞金医院血液科，上海 201800 Department of Hematology, Ruijin Hospital Affiliated to Shanghai Jiao Tong University School of Medicine, Shanghai 201800, China

## Abstract

为探讨贝林妥欧单抗在成人急性B淋巴细胞白血病（B-ALL）患者中的疗效及安全性，纳入2022年6月至2024年4月瑞金医院血液科收治并采用贝林妥欧单抗治疗的16例B-ALL患者的临床资料并进行回顾性分析。16例患者中，10例为复发/难治B-ALL，6例为新诊断的Ph^-^ B-ALL患者。10例复发/难治患者在使用贝林妥欧单抗1个疗程后有8例患者达到完全缓解且微小残留病（MRD）阴性。6例新诊断患者在初始诱导化疗后序贯贝林妥欧单抗治疗，1个疗程后复查骨髓MRD均为阴性。其中有4例患者顺利完成异基因造血干细胞移植并持续缓解。提示贝林妥欧单抗对于复发/难治B-ALL、新诊断B-ALL患者均有较高的缓解率，为后续桥接移植和延长患者生存时间提供了可能，而且不良反应可控。

## Abstract (en)

This study aimed to investigate the efficacy and safety of blinatumomab in adult patients with acute B-lymphoblastic leukemia（B-ALL）by conducting a retrospective analysis of the clinical data from 16 patients with B-ALL receiving blinatumomab at the Ruijin Hospital, Shanghai Jiao Tong University School of Medicine, from June 2022 to April 2024. Among the 16 patients, 10 were classified as relapsed/refractory B-ALL and 6 were newly diagnosed Ph^-^ B-ALL. Of the 10 patients with relapsed/refractory B-ALL, 8 achieved complete remission（CR）and minimal residual disease（MRD）negativity after one blinatumomab treatment cycle. In the 6 newly diagnosed patients, the bone marrow MRD was negative after one blinatumomab treatment cycle after initial induction chemotherapy followed by sequential blinatumomab treatment. Among them, four completed allogeneic hematopoietic stem cell transplantation and continuously maintained CR. This indicates that blinatumomab exhibits a high remission rate in both patients with relapsed/refractory and newly diagnosed B-ALL, thereby providing the possibility of bridging to transplantation and extending patient survival, with manageable adverse reactions.

贝林妥欧单抗作为第一个得到批准用于临床的双特异性T细胞连接器（BiTE），在急性B淋巴细胞白血病（B-ALL）患者群体中展示了积极的治疗潜力。本项研究对贝林妥欧单抗治疗10例复发/难治B-ALL患者和6例新诊断B-ALL患者的疗效和不良反应进行了回顾性分析。

## 病例与方法

1. 病例：回顾性分析2022年6月至2024年4月瑞金医院北部院区血液科收治并采用贝林妥欧单抗治疗的16例B-ALL患者的临床资料。所有患者均采用MICM（细胞形态学、免疫学、细胞遗传学和分子遗传学）诊断模式，诊断参照WHO 2022（第5版）标准。

2. 方法：收集患者的年龄、性别、血常规、骨髓细胞形态学、免疫学分型、细胞遗传学、分子生物学、化疗方案、微小残留病（MRD）、并发症、贝林妥单抗方案、生存情况等资料。根据用药前后骨髓缓解情况分析其疗效，同时根据用药期间并发症分析其安全性。参照《中国成人急性淋巴细胞白血病诊断与治疗指南（2021年版）》和相关指南[1]–[2]，骨髓完全缓解（CR）定义为：骨髓细胞形态学原始细胞小于5％，外周血中无原始细胞。骨髓复发指ALL CR后外周血或骨髓中再次出现原始细胞（比例大于5％）或出现髓外病变。难治指诱导治疗结束未能取得CR或CR伴血细胞不完全恢复（CRi）；第1次CR后6个月内复发；第2次CR后6个月以上复发且经诱导治疗未能缓解；2次及以上复发；出现髓外病变。

3. 贝林妥欧单抗具体用药剂量及时间、周期：患者24 h持续静脉滴注贝林妥欧单抗，第1天至第7天9 µg/d，第8天至第14天或第28天28 µg/d。治疗第1、8天予以地塞米松20 mg预处理。

4. 不良反应评价：根据Lee等[3]提出的细胞因子释放综合征（CRS）分级标准，对本研究中贝林妥欧单抗使用过程中产生的CRS进行评估。

5. 随访及观察指标：随访终点为2024年4月26日或者患者死亡时间。中位随访时间为11.8（1.2～27.0）个月。采用住院和门诊病历检索结合电话随访。观察指标包括生存情况、联合移植或后续治疗的情况、疗效评估以及不良反应。

6. 统计学处理：描述性分析，计数资料采用例数及百分比表示，非正态分布的计量资料采用中位数（范围）表示。

## 结果

1. 患者一般临床资料：共16例B-ALL患者使用贝林妥欧单抗，其中男11例，女5例。初诊时中位年龄41（17～73）岁。16例患者中，在贝林妥欧单抗治疗前，有10例属于复发/难治B-ALL，6例为新诊断的Ph^-^ B-ALL患者，这6例患者经初始诱导±Hyper-CVAD A方案化疗后达到CR，并为桥接异基因造血干细胞移植使用贝林妥欧单抗（表1）。

**表1:** 16例急性B淋巴细胞白血病（B-ALL）患者的临床特征

例号	性别	年龄（岁）	疾病状态	染色体核型	基因	既往治疗
1	男	34	复发	46,XY,del（9）（p21）/46,XY	N-RAS、PAX5突变	VDCP、A/B方案、HSCT
2	女	65	复发	正常核型	BCR::ABL1（p210）融合基因、BCOR、ABL1 T315I突变	VDCP、达沙替尼、奥雷巴替尼、维奈克拉
3	男	34	新诊断诱导化疗后	正常核型	TP53、NF1突变	VDCLP、A方案
4	男	58	难治	正常核型	ADD1::JAK2融合基因	VDCLP、卢可替尼+维奈克拉、贝博萨
5	男	47	难治	44～45,X,−Y［cp10］	KRAS、ARID1A、SETD2、JAK1突变	VDCP、VDLP、B方案、奥加伊妥珠单抗、MTX
6	男	17	新诊断诱导化疗后	正常核型	CDKN2A、CDKN2B突变	VDCLP
7	女	46	新诊断诱导化疗后	46～48,XX,+1,del（2）（p16）,+16［cp4］/46,XX	CREBBP::ZNF384融合基因、KRAS突变	VDCLP、A方案
8	男	18	难治	44～45,X,−Y,t（1;9）（p13;p12）［cp9］/46,XY	CDKN2A::TGFBR3融合基因	VDCP
9	女	33	新诊断诱导化疗后	正常核型	NRAS、U2AF1、PHF6突变	VDCLP、A方案
10	女	54	难治	骨髓干抽，未做	BCR::ABL1（p210）融合基因	VDCP、A方案
11	男	35	新诊断诱导化疗后	正常核型	ASXL1、PHF6、KDM6A突变	VDCLP
12	女	34	难治	42～46,X,−X,−6,del（8）（q13）,−12,+2～3mar［cp9］/46,XX	KRAS突变、PVT1::IGLL5融合基因	VDCLP
13	男	61	复发	正常核型	BCR::ABL1（p190）融合基因；NRAS、PAX5、WT1突变	VDCP、伊马替尼、氟马替尼、维奈克拉、达沙替尼
14	男	73	难治	46～68,XY,add（1）（p36）,−4,−7,−14,+4～8mar［cp7］/46,XY	TP53突变	VDCP
15	男	71	难治	43～45,XY,−20［cp9］/46,XY	PAX5、FLT3、CBL突变	VDCP、A方案
16	男	36	新诊断诱导化疗后	正常核型	RNF213::SLC26A11融合基因、IL7R突变	VDCP、A方案+培门冬酶

**注** VDCP：长春地辛+去甲氧柔红霉素+环磷酰胺+泼尼松；A方案：环磷酰胺+长春地辛+表柔比星+地塞米松；B方案：甲氨蝶呤+阿糖胞苷；HSCT：造血干细胞移植；VDCLP：长春地辛+去甲氧柔红霉素+环磷酰胺+培门冬酶+泼尼松；VP：长春地辛+泼尼松；MTX：甲氨蝶呤

2. 疗效情况：截至末次随访时，16例患者中有13例生存，3例死亡。每例患者的治疗反应和生存情况见图1。

**图1:**
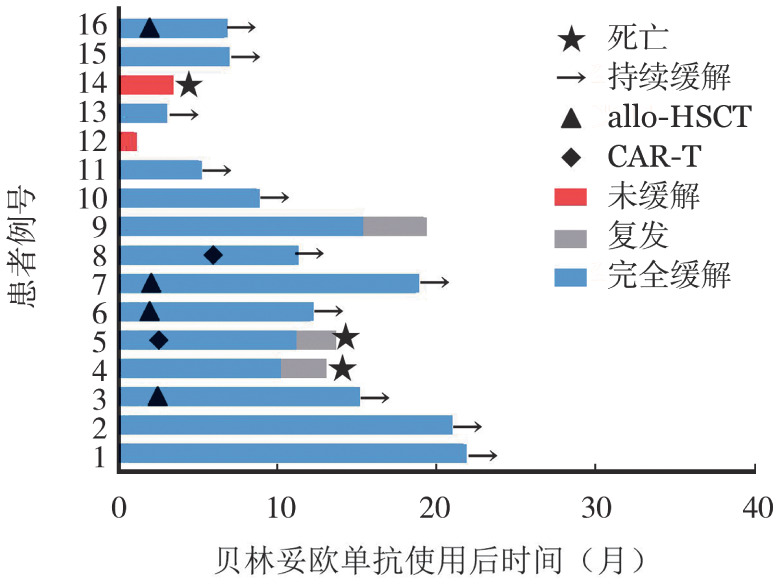
16例急性B淋巴细胞白血病患者治疗反应和生存情况的泳道图

6例新诊断B-ALL患者（例3、6、7、9、11、16），经初始VDC（L）P方案诱导±Hyper-CVAD A方案化疗后达到CR，其中例16的骨髓流式细胞术MRD为0.08％，其余患者为骨髓流式细胞术MRD阴性CR患者。予以贝林妥欧单抗治疗1个疗程后复查骨髓流式细胞术MRD均为阴性。该6例患者中已有4例患者行异基因造血干细胞移植，例11正在等待移植。例9因经济原因未接受移植，贝林妥欧单抗治疗1个疗程后至当地医院计数化疗。截至随访结束时，该6例新诊断患者中除例9已骨髓复发外，其余5例患者骨髓均为CR、MRD阴性。

10例复发难治患者中，有3例复发患者，例2、例13为Ph^+^B-ALL,VDCP方案诱导化疗CR后予以VP方案+酪氨酸激酶抑制剂（TKI）治疗直至复发。例13为骨髓复发，例1、例2为骨髓加中枢神经系统复发，该3例患者复发时骨髓原始细胞均在50％以上，故予以化疗或地塞米松降低肿瘤负荷，例1在单抗治疗前骨髓原始细胞为55％，予以Hyper-CVAD A方案化疗1个疗程后原始细胞降至2％；例2在单抗治疗前骨髓原始细胞为51.5％，予以奥雷巴替尼+维奈克拉治疗2周后原始细胞降至1.5％；例13在单抗治疗前骨髓原始细胞为92％，予以达沙替尼+维奈克拉治疗2周后原始细胞降至1％。肿瘤负荷降低后立即予以贝林妥欧单抗治疗，治疗1个疗程后达骨髓CR，骨髓及脑脊液流式细胞术MRD均转阴。7例难治患者在使用单抗后仅2例未达CR，其余患者均达到CR且流式细胞术MRD阴性（表2）。

**表2:** 接受贝林妥欧单抗治疗的16例B-ALL患者疗效评估及随访

例号	用贝林妥欧前骨髓	用贝林妥欧前脑脊液MRD	用贝林妥欧后骨髓	用贝林妥欧后脑脊液MRD	贝林妥欧后后续治疗	末次骨髓情况	是否存活
1	涂片原始细胞55％，MRD 38％	原始细胞26.55％	CR，MRD阴性	阳性	单抗	CR	是
2	涂片原始细胞51.5％，MRD 39.5％	阳性	CR，MRD阴性	阴性	单抗+奥雷巴替尼	CR	是
3	CR，MRD阴性	阴性	CR，MRD阴性	阴性	allo-HSCT	CR	是
4	CR，MRD 0.004％	阴性	CR，MRD阴性	阴性	化疗	复发	否
5	CR，MRD 0.01％	阴性	CR，MRD阴性	阴性	CAR-T	复发	否
6	CR，MRD阴性	阴性	CR，MRD阴性	阴性	allo-HSCT	CR	是
7	CR，MRD阴性	阴性	CR，MRD阴性	阴性	allo-HSCT	CR	是
8	涂片原始细胞8％，MRD 0.31％	阴性	CR，MRD阴性	阴性	CAR-T	CR，MRD阴性	是
9	CR，MRD阴性	阴性	CR，MRD阴性	阴性	化疗	复发	是
10	NR，涂片原始细胞占35.5％，MRD 1.09％	未做	CR，MRD阴性	阳性	单抗+尼洛替尼	CR，MRD阴性	是
11	CR，MRD阴性	阴性	CR，MRD阴性	阴性	无	CR，MRD阴性	是
12	NR，原始细胞占20％，MRD 27.34％	未做	NR（涂片原始细胞66％）	未做	无	NR	是
13	涂片原始细胞92％，MRD 81.86％	阴性	CR，MRD阴性	阴性	化疗	CR，MRD阴性	是
14	NR，原始细胞83％	未做	NR（涂片原始细胞98％）	未做	化疗	NR	否
15	涂片原始细胞36％，MRD 17.16％	阴性	CR，MRD阴性	阴性	化疗	CR	是
16	CR，MRD 0.08％	阴性	CR，MRD阴性	阴性	allo-HSCT	CR	是

**注** CR：完全缓解；NR：未缓解；MRD：流式细胞术微小残留病；allo-HSCT：异基因造血干细胞移植；CAR-T：嵌合抗原受体T细胞

3. 不良反应：用药过程中，发生1级CRS 2例，其临床表现为发热、Ⅳ度骨髓抑制，暂停用药好转后再次用药，患者可耐受并完成疗程。1例患者在加量至28 µg/d后第2天发生免疫效应细胞相关神经毒性综合征（ICANS），暂停抗体，予以地塞米松后好转，减量至9 µg输注至第14天。

## 讨论

自2010年初以来，靶向CD19和CD22的双特异性抗体（如贝林妥欧单抗）在B-ALL中的疗效被深入研究和探索。贝林妥欧单抗可与B细胞表面的CD19和T细胞表面的CD3结合，激活T细胞，引起CD19阳性的原始细胞裂解[4]–[6]，其疗效优于化疗，同时用于CR后MRD清除治疗时可延长患者的总生存（OS）期[7]–[8]。

在Ph阴性或阳性B-ALL患者中，贝林妥欧单抗与化疗或TKI联合应用的研究显示良好疗效。ALCANTARA研究[9]纳入45例成年Ph^+^ B-ALL患者，研究表明贝林妥欧单抗单药治疗TKI失败的Ph^+^ B-ALL患者具有良好的抗白血病活性，且耐受性良好。Topp等[10]汇总分析了两项Ⅱ期临床试验结果，数据表明贝林妥欧单抗治疗复发难治Ph^-^ B-ALL患者中位OS期为7.5个月，而CR后桥接allo-HSCT的患者中位OS期为18.1个月。本研究结果显示，截至末次随访时，16例患者中有13例生存，OS率为81％，3例死亡病例均为难治B-ALL。其中例14存在TP53基因突变，为原发耐药患者。7例难治患者在使用贝林妥欧单抗后的骨髓CR率为71％，MRD转阴率71％，另外3例复发患者骨髓CR率为100％，MRD转阴率100％。治疗前仅MRD阳性患者转阴率为100％。对于使用单抗前骨髓原始细胞比例较高的患者，我们预先予以小剂量化疗降低肿瘤负荷。化疗后均序贯予以单抗治疗。我们推测，化疗序贯单抗可使复发患者取得更好的疗效，且仅MRD阳性的患者转阴率更高，也能避免严重的CRS。

各国学者还将贝林妥欧单抗加入到新诊断ALL患者的治疗方案中，取得了较好的疗效，一项真实世界研究强调应考虑在巩固治疗而非复发时使用贝林妥欧单抗。另外几项主要研究显示了贝林妥欧单抗在新诊断B-ALL一线方案中的良好作用。美国M.D. Anderson癌症中心进行了一项单臂单中心Ⅱ期临床试验，纳入38例年轻新诊断Ph阴性B-ALL患者，将hyper-CVAD化疗方案联合序贯贝林妥欧单抗治疗，治疗有效且安全，3年无复发生存率为73％，CR率为100％[11]。Boissel等[12]证实了在巩固及维持期使用贝林妥欧单抗能使新诊断Ph^-^ ALL患者获得较高的生存率。另有一项研究对29例老年新诊断B-ALL患者应用贝林妥欧单抗作为诱导及巩固治疗，CR率为66％，3年总生存率预期为37％[13]。近期国内一项多中心、回顾性真实世界队列研究分析了20例新诊断B-ALL患者，采用减剂量化疗序贯贝林妥欧单抗的治疗方案，实现了化疗和免疫治疗之间的良好平衡，既最大限度地减少了化疗相关的并发症，又提高了诱导治疗的疗效[14]。

在此，我们回顾分析了本研究中的6例使用贝林妥欧单抗的新诊断B-ALL患者。以上6例患者在VDCLP诱导化疗±Hyper-CVAD A方案化疗后达CR，后序贯贝林妥欧单抗治疗。截至随访结束时，有5例患者均为MRD阴性CR，且已有4例患者完成异基因造血干细胞移植，移植过程顺利。其中1例复发患者为贝林妥欧单抗治疗1个疗程后改为普通化疗巩固治疗的患者，这也印证了贝林妥欧单抗桥接移植相对于化疗的优越性。

综上所述，本研究结果提示贝林妥欧单抗对于复发/难治B-ALL、MRD阳性B-ALL、新诊断B-ALL患者均有较高的缓解率，为后续桥接移植和延长患者生存时间提供了可能，而且不良反应可控。本研究样本量较少且是回顾性研究，我们希望继续通过更加严谨的前瞻性研究，去进一步巩固和丰富新的免疫靶向治疗手段在国内B-ALL患者中疗效的循证医学证据。
